# Human Ubc9 Is Involved in Intracellular HIV-1 Env Stability after Trafficking out of the Trans-Golgi Network in a Gag Dependent Manner

**DOI:** 10.1371/journal.pone.0069359

**Published:** 2013-07-08

**Authors:** Christopher R. Bohl, Levon G. Abrahamyan, Charles Wood

**Affiliations:** Nebraska Center for Virology and the School of Biological Sciences, University of Nebraska, Lincoln, Lincoln, Nebraska, United States of America; Institute of Molecular and Cell Biology, Biopolis, United States of America

## Abstract

The cellular E2 Sumo conjugase, Ubc9 interacts with HIV-1 Gag, and is important for the assembly of infectious HIV-1 virions. In the previous study we demonstrated that in the absence of Ubc9, a defect in virion assembly was associated with decreased levels of mature intracellular Envelope (Env) that affected Env incorporation into virions and virion infectivity. We have further characterized the effect of Ubc9 knockdown on HIV Env processing and assembly. We found that gp160 stability in the endoplasmic reticulum (ER) and its trafficking to the trans-Golgi network (TGN) were unaffected, indicating that the decreased intracellular mature Env levels in Ubc9-depleted cells were due to a selective degradation of mature Env gp120 after cleavage from gp160 and trafficked out of the TGN. Decreased levels of Gag and mature Env were found to be associated with the plasma membrane and lipid rafts, which suggest that these viral proteins were not trafficked correctly to the assembly site. Intracellular gp120 were partially rescued when treated with a combination of lysosome inhibitors. Taken together our results suggest that in the absence of Ubc9, gp120 is preferentially degraded in the lysosomes likely before trafficking to assembly sites leading to the production of defective virions. This study provides further insight in the processing and packaging of the HIV-1 gp120 into mature HIV-1 virions.

## Introduction

Gag (Pr55) is the predominant structural protein of HIV-1 and drives the assembly of virus-like particle in the absence of other viral proteins. Gag is translated on free ribosomes in the cytoplasm where it then traffics through an ill-defined route to the sites of assembly on the plasma membrane (PM). Gag multimerizes and initiates the budding process as the immature procapsid assembles. Gag is also involved in the selective packaging of the viral genome, various cellular host factors, and incorporation of the viral protease and replication enzymes through co-assembly with Gag-Pol fusion proteins. The replication enzymes are then cleaved into the mature forms along with Pr55 during virion maturation events (Gag function reviewed in [1–3]).

The envelope glycoprotein is an essential viral structural protein that is incorporated into the assembling virions, and is responsible for virion binding and entry into a target cell. Envelope is expressed as an immature precursor protein (gp160) within the lumen of the endoplasmic reticulum (ER). The gp160 is highly glycosylated and forms trimers within the ER. The sugar moieties are extensively modified as it is trafficked to the Golgi complex where gp160 undergoes proteolytic cleavage into gp120 and gp41. Cleavage of gp160 is believed to be mediated by furin, a host encoded protease located in the trans-Golgi network (TGN). After cleavage, the mature fusion competent Env is trafficked through the unregulated secretory pathway to the PM where it is incorporated into the assembling virions. Alternatively, the Env in the PM can be endocytosed and recycled back to the TGN [4–6]. Currently, the mechanism leading to Env incorporation is still not fully understood. However, there is evidence to suggest that under normal conditions, sequences within the cytoplasmic tail (CT) of Env interact with the matrix (MA) domain within Gag, resulting in the specific incorporation of the mature fusion competent Env (reviewed in [7–10]).

HIV-1 assembly predominantly takes place at the plasma membrane [11,12]. The assembly of a fully infectious virion requires a coordinated effort of viral and host factors to co-traffic the essential components to the sites of assembly, and in some cases excluding factors, such as tetherin and APOBEC3G [13,14]. A large number of host factors have been identified to interact with viral proteins to direct their correct trafficking to the assembly sites [15]. These include KIF3A, KIF3C, KIF4, POSH, SOCS1, Rab9 and Rab11a, AP-1, AP-2, AP-3, Tip47, staufen, GGA and ARF, SNARE proteins, ABCE1, CD81, phosphatidylinositol (4,5) bisphosphate, lipid rafts, clathrin, TSG101, and AIP1 [4,6,16–37]. The exact timing and location of where most these proteins interact during the viral assembly pathway are still not fully understood.

One important knowledge gap is our understanding of when and where Gag and Env first interact during the assembly process to facilitate Env incorporation into the released virions. Various mechanisms have been proposed for Env incorporation. Under normal conditions, either direct or indirect interactions between amino acids within MA and the cytoplasmic tail of Env result in the selective incorporation of the viral Env (reviewed in 7–10. Confocal microscopy data examining the site of co-localization between Gag and Env suggested that Gag and Env interact at the PM [27,38]. However various lines of evidence have suggested that the interaction may occur before virion assembly at the PM, either through direct or indirect interactions that result in the active incorporation of mature Env into the assembling virions [27,39–44]. Regardless of where Gag and Env first interact, there is evidence to suggest that both Gag and Env influence the stability and trafficking of each other [5,38–40,45–48], which is important for the assembly of an infectious virion.

We have previously reported that the host factor Ubc9 interacts with Gag and plays an important role in the HIV-1 assembly pathway [49]. Ubc9 is an E2 SUMO conjugase that post-translationally modifies target proteins and alter their function by the addition of SUMO (reviewed in 50,51. However, a growing list of proteins have been found to interact with Ubc9 to result in their altered function without being targets for SUMOylation [52–59]. Ubc9 and SUMOylation have been shown to play important roles in the replication cycles of both DNA and RNA viruses, and some of these viruses deregulate the host SUMOylation pathway to enhance their replication [60–62]. We previously reported that knockdown of the endogenous Ubc9 expression in HIV-1 producer cells results in the production of defective virions. The defect in infectivity was due to decreased levels of mature intracellular Env and reduced Env incorporation into the released virions. Interestingly, the decreased intracellular Env levels in Ubc9 knockdown cells were dependent upon Gag expression, as intracellular Env levels were not affected in the absence of Gag and Ubc9 expression. Experiments with the trans-dominant negative Ubc9 (C93A), lacking the SUMO conjugase activity, have suggested that the enzymatic activity of Ubc9 did not play a role in Gag dependent reduction of intracellular Env levels [49].

In order to further understand the role of Ubc9 and how its depletion leads to the decreased levels of mature intracellular Env to result in production of defective virions, we have systematically examined which step(s) in the virus assembly pathway might be affected by Ubc9 knockdown and lead to a reduction in Env incorporation. We found that in Ubc9 knockdown cells, the decreased levels of intracellular mature Env is not due to an increase in gp160 instability in the ER, or a defect in subsequent trafficking to the TGN compartment. We found that mature Env is selectively targeted for degradation, possibly via the lysosomal pathway after it is processed from gp160 and transported out of the TGN. We also found that there were decreased levels of Gag and mature Env in the PM and lipid rafts. Taken together, our findings suggest that in Ubc9 knockdown cells, there is mistrafficking of Gag and Env to microdomains of the PM known to be important for virion assembly, and the degradation of mature Env likely occurred before trafficking to the PM.

## Materials and Methods

### Ethics statement

Certificate of Exemption approval was obtained from the University of Nebraska-Lincoln Institutional Review Board under project ID 10956.

### Cell culture and transfection

293T cells were obtained from the American Type Culture Collection, they were cultured and transfected with control and Ubc9 siRNA as previously reported [49]. TZM-bl indicator cells were obtained through the NIH AIDS Research and Reference Reagent Program, Division of AIDS, NIAID, NIH: TZM-bl from Dr. John C. Kappes, Dr. Xiaoyun Wu and Tranzyme Inc.

### Plasmids

The infectious HIV-1 proviral clone pNL4-3 was obtained from the AIDS Research and Reference Reagent Program, Division of AIDS, NIAID, NIH: pNL4-3 from Dr. Malcolm Martin [63]. The cleavage defective envelope mutant (pNL4-3 MUT 511) was a kind gift from Dr. Valerie Bosch and has been previously described [64].

### Metabolic labeling, immunoprecipitation and Endoglycosidase treatments

Metabolic labeling experiments were carried out as previously reported with differences in labeling times with [^35^S] methionine/cysteine (>1,000 Ci/nMol; NEN) [49]. Briefly, transfected and untransfected 293T cells were labeled for 30 minutes, 1 hour, or 4 hours with 300 µCi, 600 µCi, or 2.0 mCi respectively. For pulse chase experiments, labeling media were removed and chased for 2 and 4 hours in complete culture media. The culture media was removed, clarified by centrifugation (13,000 RPM for 2 min), and adjusted to 1X lysis buffer [65]. Cells were lysed with 1X lysis buffer containing Halt protease inhibitors cocktail (Pierce) and clarified by centrifugation. Viral proteins were immunoprecipitated using AIDS patient sera and Ultralink A/G beads (Thermo Scientific). The beads were washed four times with lysis buffer, boiled for 5 minutes in 0.04% SDS and 200mM 2-mercapitoethanol [66]. The solublized proteins were divided into two equal aliquots and treated with Protein N-Glycosidase F (PNGase F) or Endoglycosidase H_f_ (Endo H_f_) (NEB) according to manufacturers suggestions for 3.5 hours, or left untreated. All samples were adjusted to 1X protein sample buffer (PSB), separated by SDS-PAGE, and visualized by phosphor imaging. Band intensities were quantified using Discovery Series Quantity One software (Bio-Rad). Alternatively, viral proteins were solublized directly from the A/G beads using 2X PSB, boiled, gel separated and examined as above.

### Inhibitor treatments

Transfected and untransfected cells were treated with various protease inhibitors: proteasome inhibitor MG132 (10 µM) and lysosome inhibitors E63d (10 µg/ml), Pepstatin A (10 µg/ml), and Leupeptin (5 µg/ml). All inhibitors were purchased from Sigma. Cells were pretreated with the appropriate inhibitors prior to metabolic labeling and maintained throughout the pulse-chase experiment. Pulse-chase samples were processed and analyzed as above.

### Unfolded protein response

Activation of the unfolded protein response was analyzed by XBP-I splicing as previously described with slight modifications [67]. Briefly, transfected and untransfected 293T cells were treated with 5mM dithiothreitol (DTT) for 3 hours or left untreated. Total RNA was extracted from 293T cells using the RNeasy mini kit (Qiagen) and treated with DNase I amplification grade (Invitrogen) according to manufacturer’s protocol. Three micrograms of RNA was reverse transcribed using oligo (dT) and SuperScript III (Invitrogen) per manufacturer’s protocol. XBP-1 cDNA was amplified using standard PCR techniques with primers that flank the splice site (5’-ccttgttgagaaccagg-3’ and 5’-ctaagactaggggcttggta-3’).

### Plasma membrane and Lipid raft/detergent resistant membrane isolation

Cells were pulse labeled with [^35^S] methionine/cysteine for 30 minutes and chased for 1.5 and 2.5 hours. At the chase times, cells were quickly cooled on ice. Medium was removed from the culture dishes and cells were collected in ice-cold sucrose buffer (16% by weight), ruptured by dounce homogenization, and followed with Benzonase treatment (50 units) for 0.5 hours. The plasma membrane was separated from membranous organelles by ultracentrifugation. Samples were centrifuged at 4°C, for 20 hours at 34,500 RPMs in a ML-130 rotor. Supernatants were carefully collected leaving behind the pellet containing cellular organelles and adjusted to 1X lysis buffer. Viral proteins were immunoprecipitated with HIV-1 patient serum, separated by SDS PAGE, and visualized by phosphorimaging using The Discovery Series Quantity One software. Lipid raft isolations were preformed as previously described, with slight modifications [68]. Transfected 293T cells were radiolabeled for 4 hours with 2.0 mCi [^35^S] methionine/cysteine. The labeling media was removed and the cells were lysed on ice with 500 µl of TNE buffer (10 mM Tris [pH 7.5], 100 mM NaCl, 10 mM EGTA) containing 0.5% Triton X-100 (TX-100) for 30 min. Lysates were collected and homogenized with 10 strokes through a 25-G needle, and then clarified by low speed centrifugation at 10,000 RPMs at 4°C for 10 minutes. Post nuclear lysates were adjusted to 60% sucrose by adding 1.5 ml of 80% sucrose TNE (w/v). The lysates were layered over 500 µl of 80% sucrose TNE, followed by 2 ml of 50% sucrose TNE, 6ml of 38% sucrose TNE, and 1.5 ml of 10% sucrose TNE. The sucrose gradients were centrifuged at 100,000 X g at 4°C for at least 18 hrs in a Beckman SW41 rotor. Eleven fractions were collected using a piston gradient fractionator (Biocomp). Densities of each fraction were determined by the refractive index of each sample. Fractions were adjusted to 1X lysis buffer, viral proteins were immunoprecipitated with pooled HIV-1 patient serum, separated by SDS-PAGE and visualized by phosphor imaging. Cellular proteins were precipitated from each fraction by methanol/chloroform/water precipitation [69], then analyzed by immunoblotting to identify which sucrose fractions contained lipid rafts (LR), detergent resistant membranes (DRM) and/or detergent soluble membranes (DSM).

### Immunoblotting

Samples were lysed in lysis buffer as previously described [49], normalized for total protein concentration by BCA assays (Pierce), or samples were directly solublized in 2X PSB. Proteins were separated by SDS PAGE, transferred to nitrocellulose membrane (GE Water & Process Technologies) and detected by immunoblotting as previously described [49], or with a LI-COR Odyssey infrared imaging system.

### Antibodies

Anti-Ubc9 (N-15) goat polyclonal antibodies (PAb), anti-Actin goat PAb, anti-Flotillin (K-19) goat PAb, anti-E-Cadherin (H-108) rabbit PAb, horseradish peroxidase (HRP)-conjugated chicken anti-goat, and HRP-conjugated donkey anti-mouse PAb were purchased from Santa Cruz Biotechnology, Inc. HIV-1 anti-gp120 goat PAb was purchased from Affinity BioReagents. Anti-human transferrin receptor monoclonal antibodies were purchased from Invitrogen. HIV-IG was obtained from the NIH AIDS Research and Reference Reagent Program. Alexa Fluor 488 conjugated goat anti-human PAb were purchased from Molecular Probes (Invitrogen). IRDye 800CW conjugated donkey anti-goat and IRDye 680LT conjugated donkey anti-rabbit PAbs were purchased from LI-COR. Pooled HIV-1 infected patient sera were obtained from a patient cohort.

### Env cell surface expression quantification

HIV-1 envelope cell-surface expression was analyzed as previously described [70]. Briefly, transfected 293T cells were harvested using PBS supplemented with EDTA and EGTA and fixed for 30 min at 4°C in 2% paraformaldehyde in PBS. To quantify Env surface expression levels, fixed cells were incubated with primary antibody (HIV-IG, NIH), washed extensively and the binding of the primary antibody to the cells was detected with Alexa Fluor-488 goat-anti-human conjugated antibody. The mean fluorescence intensity (MFI) and percent of fluorophore positive (Env-expressing) cells were detected with the FACS Calibur system (BD Biosciences). For each treatment the geometrical mean fluorescence intensity value for the control stained population (mock-transfected cells) was subtracted from the MFI value of the positively stained sample. The MFI values and percentage of Env-positive cells in siRNA transfected samples are expressed as percentages of the values found in cells transfected with only pNL4-3.

## Results

### No evidence for endoplasmic reticulum associated degradation (ERAD), or activation of the unfolded protein response (UPR) as a factor in gp120 stability in Ubc9-depleted cells

**Figure 1:**
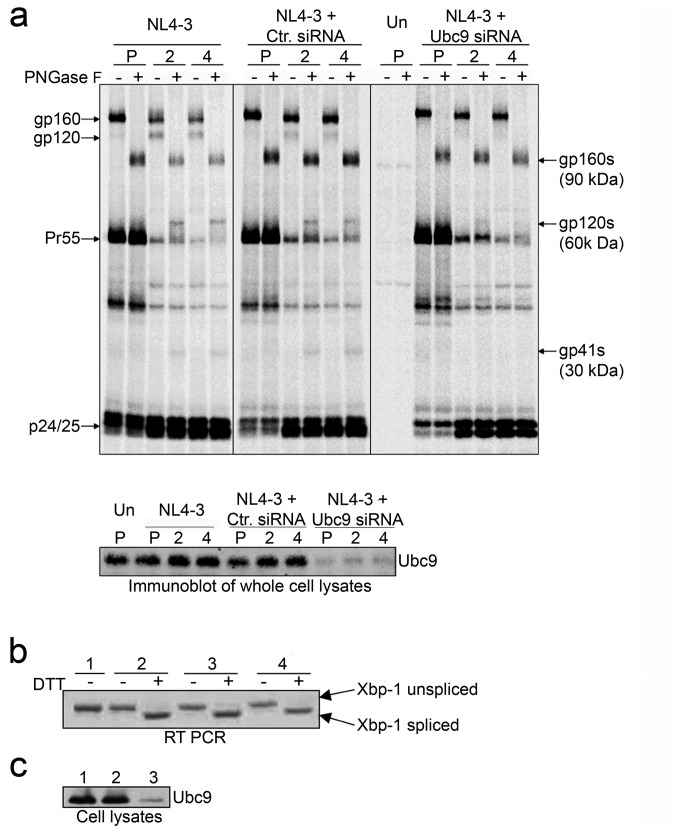
Decreased Env stability in Ubc9 knockdown cells is not due to endoplasmic reticulum stress responses. (a) Molecular weights of deglycosylated Env proteins in the presence or knockdown of Ubc9 expression. 293T cells were transfected with pNL4-3 alone, or in combination with either Ctr. siRNA or Ubc9 siRNA, or left untransfected (Un). Cells were pulse (P) labeled with [^35^S] methionine/cysteine for 1 hour and chased for 2 and 4 hours. Cell associated viral proteins were solublized and immunoprecipitated with pooled AIDS patient sera, split equally, and incubated for 3.5 hours at 37°C in the presence, or absence of N-glycosidase F (PNGase F). Samples were separated by SDS PAGE and visualized by phosphorimaging using The Discovery Series Quantity One software. A representative over-exposed gel is shown so that partially Endo H_f_ resistant Env can be more easily visualized. The identity and position of PNGase F untreated viral proteins are labeled on the left of the gel. Deglycosylated, PNGase F sensitive, viral proteins are labeled on the right and are denoted with a (s) to identify the position of gp160s, gp120s, and gp41s in the gel after PNGase F treatment. (b) Unfolded protein response activation state in the presence or knockdown of Ubc9 expression. Untransfected (1), NL4-3 alone (2), NL4-3 + Ctr. siRNA (3), NL4-3 + Ubc9 siRNA (4). Total RNA was extracted from transfected 293t cells that were either treated with 5mM DTT for 3 hours to induce UPR, or left untreated. Total mRNA was reverse transcribed with oligo (dT) followed by PCR amplification of XBP-1 cDNA using primers that flank an alternative splicing site with in the XBP-1 mRNA. (c) Immunoblot of Ubc9 expression in whole cell lysates. Untransfected (group 1), Ctr. siRNA (group 2), and Ubc9 siRNA (group 3).

To examine the possibility that in the absence of Ubc9 the decrease in intracellular gp120 could be due to an increase in gp160 degradation in the endoplasmic reticulum (ER), we examined two major ER degradation pathways (ERAD and UPR). The ERAD is an ER based pathway that degrades misfolded proteins. Proteins targeted by ERAD are partially deglycosylated, exported out the ER, and targeted for degradation in a proteasome dependent manner (reviewed in 71–73. It has been previously demonstrated that treatment of Env expressing cells with GPG-NH_2_ causes Env to undergo ERAD, which results in gp160 migrating at a molecular weight (MW) similar to wild type gp120 due to gp160 partial deglycosylation [67,74]. If a population of gp160 was partially deglycosylated via ERAD, it could affect the identification and quantitation of the gp160 and gp120 levels in the Ubc9 knockdown cells. Even though our previous study analyzing transfected cell lysates at steady state, using monoclonal antibodies that were specific for gp120 and gp41, did not suggest that ERAD was occurring [49], we could not rule out the possibility that a portion of the 120 kDa proteins observed could in fact be gp160 undergoing ERAD in our pulse chase assays. Therefore, to examine this possibility we determined the MW of deglycosylated viral glycoproteins after treatment with PNGase F (Figure 1a), which hydrolizes nearly all forms of N-linked glycans [75]. The viral glycoprotein was expressed as gp160 during the pulse-labeling period. During the chase, gp120 could be detected in cells transfected with pNL4-3 alone, or in combination with control siRNA. As expected, cells transfected with Ubc9 siRNA contained less gp120. When the lysates from the pulse period were treated with PNGase F, the band corresponding with glycosylated gp160 disappeared and a band with a MW of approximately 90 kDa was observed. This 90 kDa band correlates with the expected MW of deglycosylated gp160. When the chase samples were treated with PNGase F, the expected 90 kDa band was detected. The band corresponding to gp120 disappeared, and a band with a MW of approximately 60 kDa appeared, which correlates to the expected weight of deglycosylated gp120. The 60 kDa band was observed throughout the chase period when samples were treated with PNGase F, and was much less evident in cells transfected with Ubc9 siRNA. A band with a MW of approximately 30 kDa also appeared in these samples, which correlates with the expected size of deglycosylated gp41. The presence of the 60 kDa band in Ubc9-depleted cell lysates treated with PNGase F indicated that the band around 120 kDa in the PNGase F untreated cell lysates was gp120, and not a partially deglycosylated forms of gp160 undergoing ERAD. More importantly, gp160 did not appear to be preferentially degraded in Ubc9 knockdown cells as compared to control cells. Over-exposed gels did not show any unique or additional bands between 90 and 60 kDa, ruling out the possibility that gp160 was undergoing degradation (data not shown).

Since ERAD is not involved, we examined if the unfolded protein response (UPR) could be involved in the decrease of intracellular gp120 through degradation of gp160. The UPR is triggered when improperly folded proteins accumulate in the ER, and is a marker for overall ER stress and cellular dysfunction. Upon UPR activation, the X-box binding protein 1 (XBP-1) mRNA is alternatively spliced, leading to a frame-shift that increases the transcriptional activity of XBP-1, and upregulation of chaperones in the ER (reviewed in 76. To examine ER stress and activation of UPR, XBP-1 splicing in Ubc9-depleted cells was determined using total cellular RNA extracted from transfected 293T cells, either treated or untreated with an UPR activating agent, dithiothreitol (DTT). A 450 bp unspliced XBP-1 mRNA product was detected in untreated cells and upon addition of DTT, a 26 bp smaller spliced XBP-1 mRNA PCR product was detected, indicating that the UPR was activated (Figure 1b). However, neither HIV-1 gene expression nor knockdown of Ubc9 by siRNA activated the UPR. Duplicate plates transfected with control siRNA, Ubc9 siRNA, or left untransfected were lysed and immunoblotted for Ubc9 and actin levels to confirm knockdown of Ubc9 (Figure 1c). Taken together, the decrease in intracellular gp120 levels did not appear to be due to enhanced gp160 instability in the ER as an outcome of Ubc9 knockdown.

### Gp160 is trafficked to the trans-Golgi network normally in the absence of Ubc9 expression

**Figure 2:**
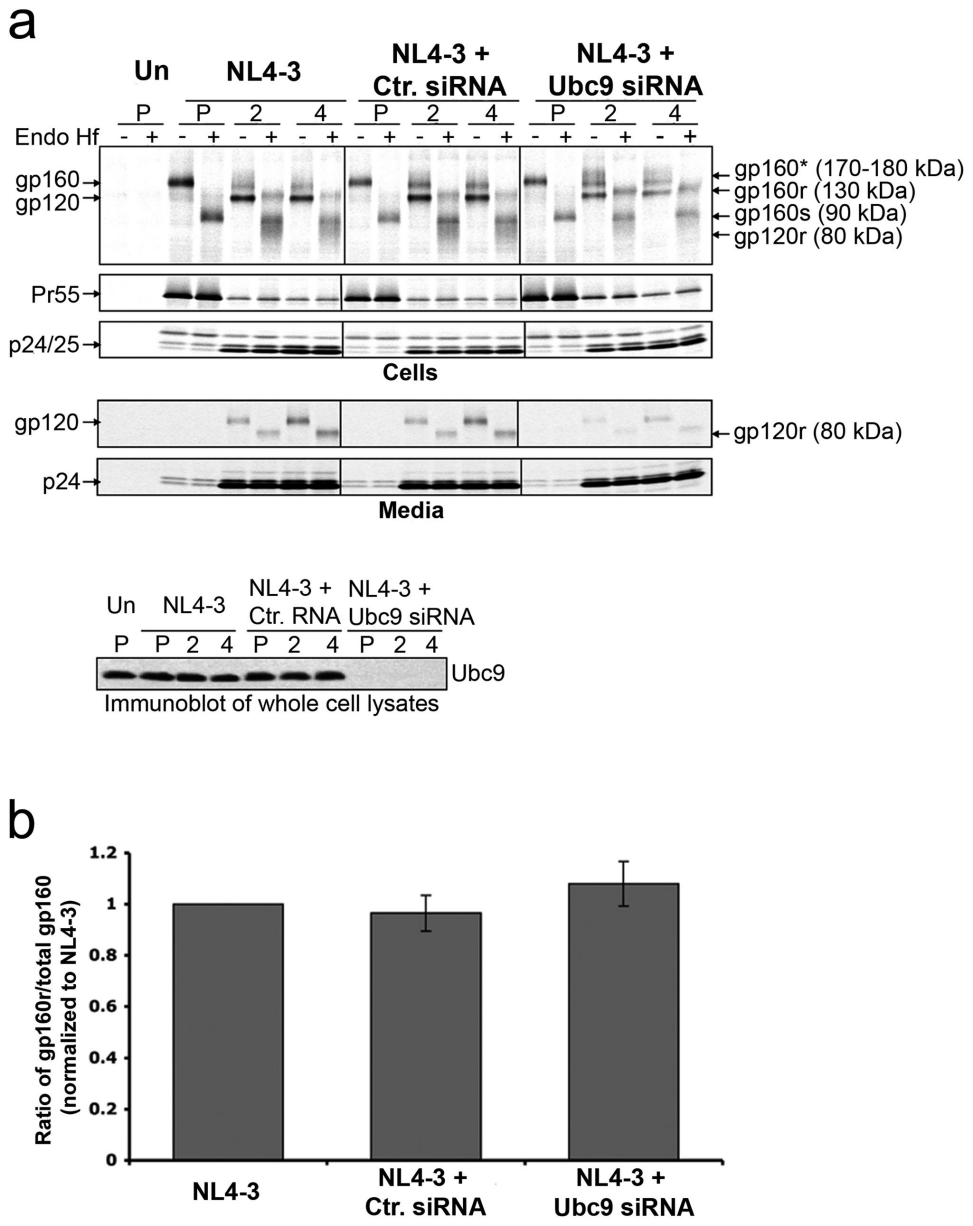
HIV-1 gp160 traffics to the Golgi network in Ubc9 knockdown cells. (a) Env trafficking through the secretory pathway. 293T cells were transfected with pNL4-3 alone, or in combination with either Ctr. siRNA or Ubc9 siRNA, or left untransfected (Un). Unlike the previous and subsequent experiments, cells were pulse (P) labeled with [^35^S] methionine/cysteine for a shorter period of time (30 minutes) and chased for 2 and 4 hours. Cell and media associated viral proteins were solublized and immunoprecipitated with pooled AIDS patient sera, split equally, and incubated for 3.5 hours at 37°C in the presence, or absence of Endo H _f_. Samples were separated by SDS PAGE and visualized by phosphorimaging using The Discovery Series Quantity One software. A representative, over-exposed gel is shown so that partially Endo H_f_ resistant Env can be more easily visualized. Viral proteins and their positions in the gel are labeled on the left. The identity of Endo H_f_, untreated viral proteins and their positions in the gel are labeled on the right. Deglycosylated Endo H_f_ sensitive forms of gp160 residing in the ER are labeled as gp160s. Partially deglycosylated, Endo H_f_ resistant forms of gp160 and gp120 that have undergone glycan modification in the TGN are labeled as gp160r and gp120r. gp160r in Endo H_f_ untreated samples is labeled as gp160*. (b) The gp160 trafficking to TGN. The ratio of gp160r/total gp160 during the 2-hour chase period, normalized to NL4-3.

With no evidence implicating ER dysfunction and associated gp160 degradation in the Ubc9-depleted cells, we next determined whether there was a defect in gp160 trafficking to the TGN, and whether cleavage and maturation could be involved in the decreased levels of intracellular gp120. To determine if Ubc9 knockdown causes defects in gp160 TGN trafficking, we tracked Env movement through the secretory pathway of Ubc9 knockdown cells and control cells by evaluating the glycosylation state of Env with a combination of pulse chase analysis and Endo H_f_ sensitivity assay (Figure 2a). HIV-1 gp160 is extensively modified with N and O-linked oligosaccharides in the ER, but they can be removed by Endo H_f_, an endoglycosidase which primarily cleaves high-mannose sugars [77]. As Env proteins trimerize in the ER and traffic through the TGN, the polyprotein oligosaccharides are substantially modified and the susceptibility to Endo H _f_ cleavage decreases. While gp160 traffics through the TGN it is cleaved into its mature forms (gp120 and gp41) ( [78–80], reviewed in 81. Unlike the previous and subsequent experiments, cells were pulse labeled with [^35^S] methionine/cysteine for a shorter period of time (30 minutes) in order to monitor and quantify a more finite population of gp160 as it is modified and trafficked through the secretory pathway. During the pulse period (P), only gp160 could be detected in control cells that were transfected with NL4-3 proviral DNA alone (Figure 2a, left panel), in cells co-transfected with NL4-3 and control siRNA (middle panel), or in cells co-transfected with NL4-3 and Ubc9 siRNA (right panel). Treatment of the pulse samples with Endo H_f_ led to the disappearance of the band of 160 kDa (gp160), and a band with an approximate MW of 90 kDa was observed. The 90 kDa band correlates with the expected MW of gp160 with its N-linked glycans removed by Endo H_f_ (gp160s). Susceptibility of gp160 to Endo H_f_ during the pulse period suggests that all the gp160 was located in the ER. As expected, during the chase period, gp160 levels decreased, and gp120 appeared along with a very diffuse band with an approximate MW of 170-180 kDa. This diffuse band correlates to a population of gp160 that traffics to the Golgi and is modified with more complex sugars [82,83]. When the chase samples were treated with Endo H_f_, gp160, gp120, and the 170-180 kDa bands disappeared, the bands with approximate MW of 130, 90, and a smear around 80 kDa were observed. The band at around 130 kDa is consistent with the migration pattern of a partially Endo H_f_ resistant gp160 (gp160r), the population of gp160 that would have trafficked out of the ER with its high-mannose sugars modified to more resistant complex sugars in the Golgi complex. The band at approximately 90 kDa is a population of gp160 that still resides in the ER (gp160s). The band/smear around 80 kDa is consistent with forms of gp120 that have undergone extensive glycosylation modifications in the Golgi and were partially resistant to Endo H_f_ (gp120r) [84].

Upon transfection with Ubc9 siRNA, or with control siRNA, levels of partially Endo H_f_ resistant forms of gp160 (gp160r) were detected beginning at two hours. Detection of similar amounts of gp160r in cells where Ubc9 expression was knocked down indicated that gp160 was able to trimerize and traffic out of the ER [85,86]. Mature Env gp120 and gp120r were detected at similar levels in cells transfected with NL4-3, or in combination with control siRNA. As expected, gp120 and gp120r were on average reduced by 50-60% in cells transfected with Ubc9 siRNA (Figure 2a). However, during the chase period, similar levels of gp160 were found to traffic out of the ER to the Golgi complex based on the amount of Endo H _f_ resistant gp160, and suggest that trafficking of gp160 to the TGN in Ubc9 knockdowns was not affected (Figure 2b). The lower intracellular gp120 in the absence of Ubc9 expression resulted in less gp120 incorporated into the virion (Figure 2a, lower panels).

### Gp120, but not gp160 is specifically degraded in the absence of Ubc9

**Figure 3:**
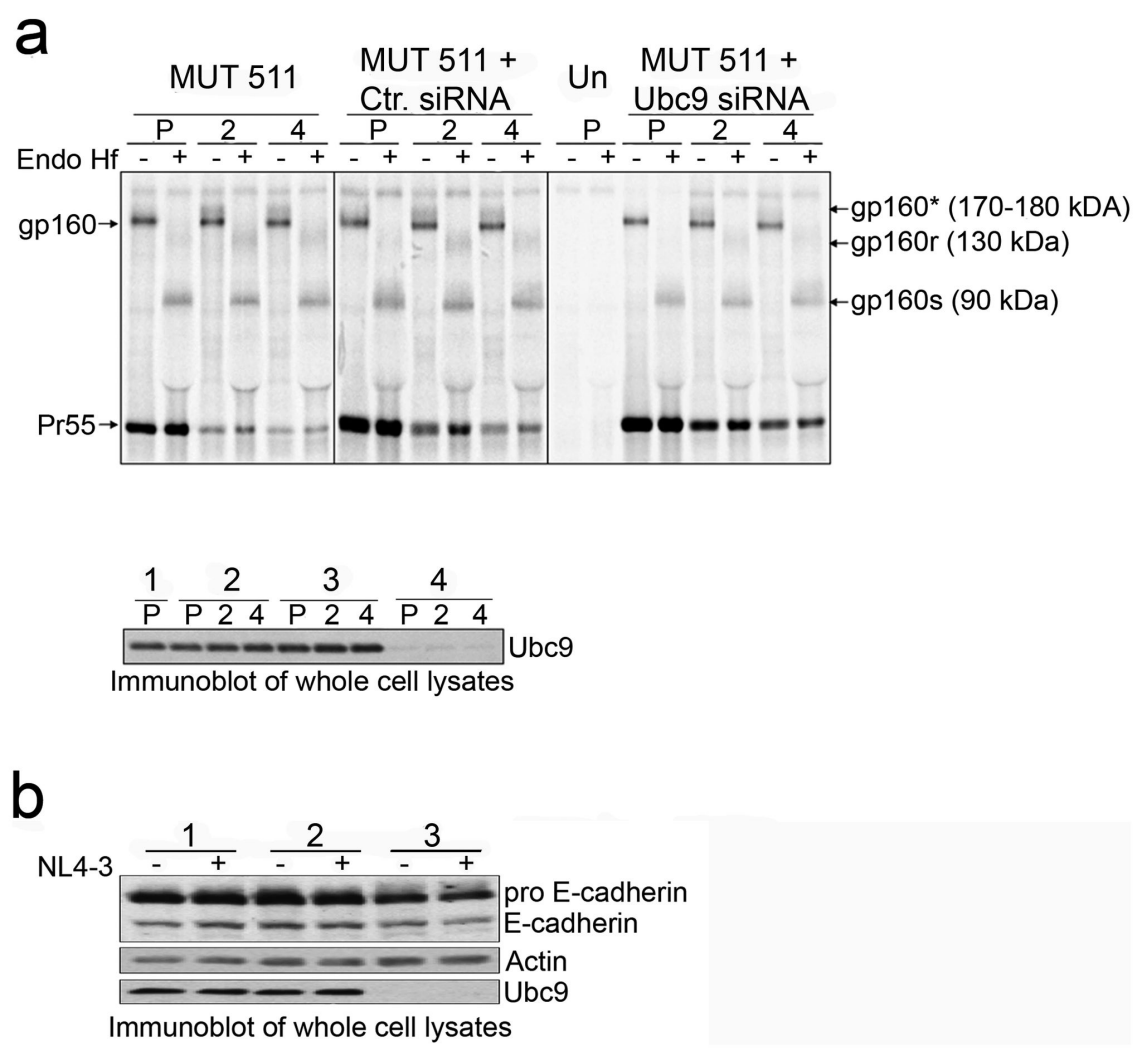
Stability of gp160 is unchanged in Ubc9 knockdown cells. (a) 293T cells were transfected with pNL4-3 MUT 511 alone, or in combination with Ctr. siRNA or Ubc9 siRNA, or left untransfected. Cells were pulse (P) labeled with [^35^S] methionine/cysteine for 1 hour, then chased for 2 and 4 hours. Samples were processed as described in previous Endo H _f_ experiment. A representative over-exposed gel is shown so that partially Endo H_f_ resistant Env can be more easily visualized. The identity of Endo H_f_, untreated viral proteins and their positions in the gel are labeled on the right. Deglycosylated Endo H_f_ sensitive forms of gp160 residing in the ER are labeled as gp160s. Partially deglycosylated, Endo H_f_ resistant forms of gp160 that have undergone glycan modification in the TGN are labeled as gp160r. These forms of gp160 denoted as gp160* in Endo H_f_ untreated samples. Lower panel: Immunoblot of Ubc9 expression in whole cell lysates; untransfected (group 1), pNL4-3 (group 2), Ctr. siRNA (group 3), and Ubc9 siRNA (group 4). (b) Furin activity is not dependent upon Ubc9 expression. 293T cells were lysed 24 hrs post transfection and cell lysates were immunoblotted with antibodies against Ubc9, Actin, and E-Cadherin. Immature, uncleaved E-Cadherin is designated as pro E-Cadherin.

Our data so far has suggested that the reduced level of intracellular gp120 in Ubc9-depleted cells was not due to defects associated with gp160 stability or trafficking. To examine whether gp120 was specifically degraded after gp160 cleavage in Ubc9 knockdown cells, we examined the stability of an Env cleavage provirus mutant (MUT 511). MUT 511 has a point mutation at position 511 (Arg to Ser) in gp160, which blocks its cleavage into gp120 and gp41. MUT 511 Env is not efficiently incorporated into virions, but still traffics to the plasma membrane [64,87]. If gp120 is specifically targeted for degradation, MUT 511 Env stability should be unchanged as it trafficks through the secretory pathway to the plasma membrane. Cells were pulse chased and treated with Endo H_f_ as in previous experiments to track MUT 511 Env stability and trafficking through the TGN (Figure 3a). As expected, only gp160 was observed, and it was not processed into gp120 and gp41 because of the mutation. During the pulse period, gp160 resided in the ER and was fully Endo H_f_ sensitive as observed earlier (Figure 3a). At the two-hour chase time a band with an approximate MW of 180 kDa appeared and persisted through the 4-hour chase period. Endo H_f_ treatment resulted in the appearance of band of 90 kDa (gp160s), a partially resistant Endo H_f_ gp160 (gp160r) as observed earlier (Figure 3a), confirming that MUT 511 gp160 was trafficking through the secretory pathway even in Ubc9 knockdown cells. Intracellular Env levels remained stable during the chase in cells that were transfected with either Ubc9 siRNA or control siRNA, indicating that the decrease in gp160 observed over time in previous experiments was due to gp160 maturation into gp120/gp41 and not due to degradation.

### Ubc9 knockdown does not affect Furin activity or endogenous E-cadherin stability

To further confirm that the increase in gp120 degradation in Ubc9 knockdown cells is specific to mature Env, and not due to a more generalized decrease in overall stability of proteins trafficking through the secretory pathway, we assayed the maturation and stability of endogenous, cellular E-cadherin. E-cadherin is a cellular, type-1 transmembrane adhesion factor and its biosynthetic pathway is very similar to that of HIV-1 Env. E-cadherin is cleaved into its functional subunits as it moves through the secretory pathway, presumably by Furin in the TNG, as it traffics to the plasma membrane (reviewed in 88,89. Equal amounts of whole cell lysates from control or Ubc9 siRNA transfected, and untransfected 293T cells were analyzed for E-cadherin expression. Pro-E-cadherin (135 kDa) and mature E-cadherin (120 kDa) can be detected in whole cell lysates transfected with Ubc9 siRNA, albeit at a slightly lower amount of total E-cadherin (immature and mature forms) as compared to control cells (on average 19-23% less). The lower E-cadherin expression was not unexpected as previous studies have shown that intracellular levels of Ubc9 regulate E-cadherin expression through miR-200b [90–93]. Even though less total E-cadherin was expressed in the Ubc9 knocked down cells, the mature E-cadherin represented 43% of total cellular E-cadherin when normalized to the levels of actin regardless of Ubc9 expression (Figure 3b). Our results indicate that in Ubc9-depleted cells, there was no evidence of a general decrease in stability of proteins such as E-cadherin while trafficking through the secretory pathway. In addition, Furin activity was not affected, as judged by pro-E-cadherin cleavage and suggest that the lower levels of gp120 in Ubc9 knockdown cells is not due to deficient Furin-mediated gp160 cleavage.

### Degradation of gp120 occurs before transport to the plasma membrane and the lipid rafts

**Figure 4:**
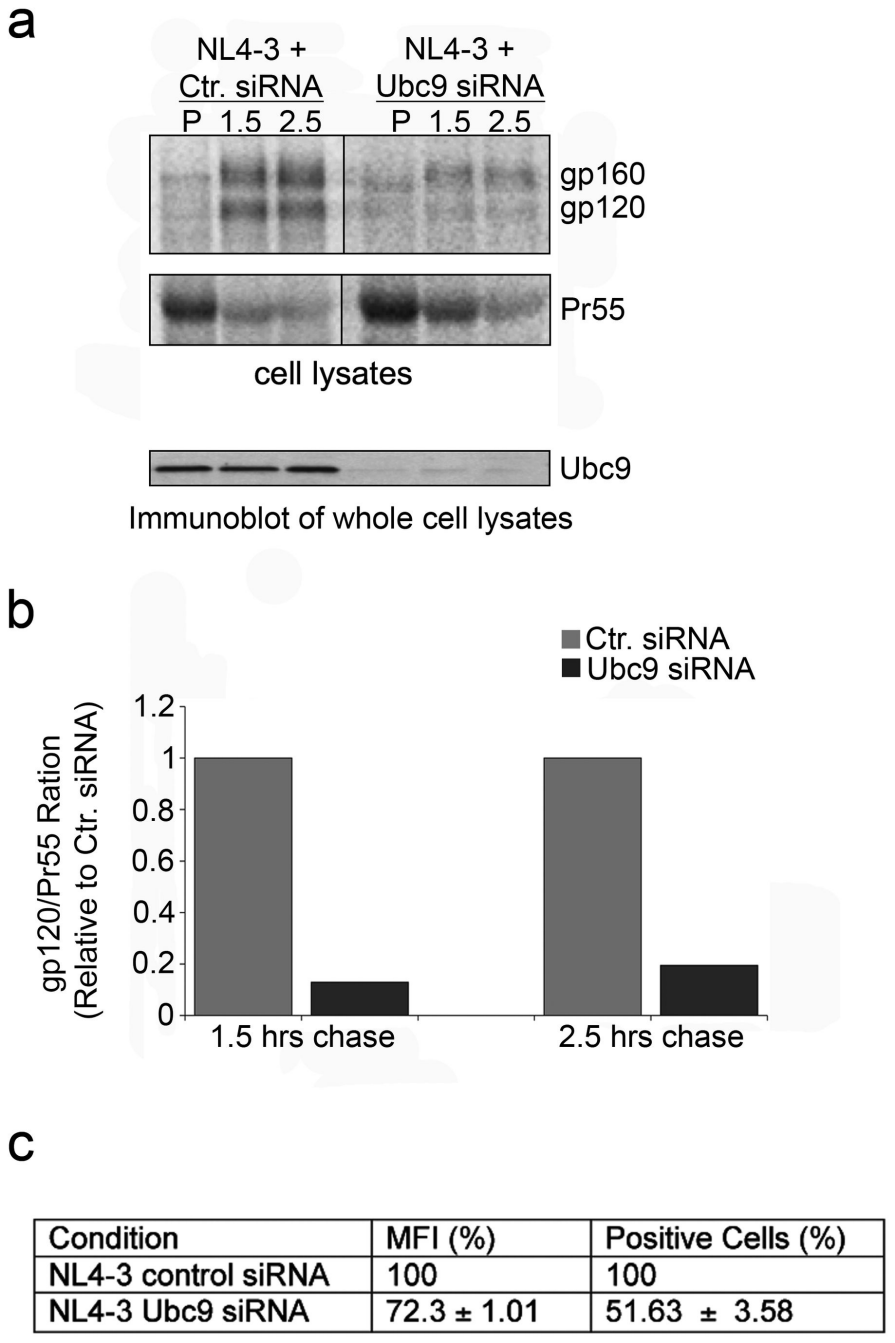
HIV-1 proteins exhibit altered trafficking to the plasma membrane in Ubc9 knockdown cells. (a) 293T cells were transfected with pNL4-in combination with either Ctr. siRNA or Ubc9 siRNA. Cells were pulse labeled with [^35^S] methionine/cysteine for 30 minutes and chased for 1.5 and 2.5 hours. At indicated times, cells were ruptured and treated with Benzonase (50 units) for 0.5 hours. Lysates were centrifuged at 4°C, for 20 hours at 34,500 RPMs in a ML-130 rotor. Supernatants were carefully collected leaving behind the pellet containing cellular organelles and adjusted to 1X lysis buffer. Viral proteins were immunoprecipitated with patient serum, separated by SDS PAGE, and visualized by phosphorimaging using The Discovery Series Quantity One software. (b) Quantitation of viral proteins associated with the plasma membrane. gp120/Pr55 ratios of the PM fraction during the 1.5 hour and 2.5 hour chase times. (c) Steady state Env surface expression. Transfected cells were resuspended in PBS, fixed, and surface Env was assayed by flow cytometry using HIV-IG and Alexa Fluor-488 goat-anti-Human conjugated Antibodies.

Our results have indicated that gp160 stability, trafficking from the ER to the TGN, and cleavage was normal. In a normal cell, after Env cleavage maturation occurs, the trimer consisting of gp120 and gp41 is trafficked to the PM. The mature Env is then either incorporated into assembling virions, or it is quickly endocytosed by the cellular endocytic machinery through interactions with adaptor protein complexes primarily through AP-2 binding [6,24,94]. It is possible that in the absence of Ubc9, gp120 stability is affected prior to reaching the PM or may be affected only after endocytosis. To further delineate when the degradation of gp120 occurred in the Ubc9 knockdown cells, we conducted experiments to examine the amount of gp120 associated with the PM and lipid rafts. To examine the levels of mature Env (gp120) associated with the PM, Ubc9-specific or control siRNA transfected 293T cells were metabolically labeled and chased for 1.5 and 2.5 hours (Figure 4a), lysed and fractionated by ultracentrifugation to separate the PM from other cellular organelles. During the pulse, very little Env proteins appeared to be associated with the PM fraction in any samples. At 1.5 and 2.5 hours, gp160 and gp120 can be readily detected in the PM fraction of cells transfected with NL4-3 alone, or with NL4-3 and control siRNA. Cells transfected with NL4-3 and Ubc9 siRNA showed an 83% decrease in the levels of gp120 associated with the PM fraction as compared to the control cells (Figure 4b). Surface expression of Env examined by confocal microscopy (data not shown) and flow cytometry at steady state levels confirmed that less Env was expressed on the cell surface in Ubc9 knockdown cells (Figure 4c). Together, these results suggested that in Ubc9 knockdown cells, less Env is present at the plasma membrane, and gp120 stability was affected prior to its insertion into the PM.

**Figure 5:**
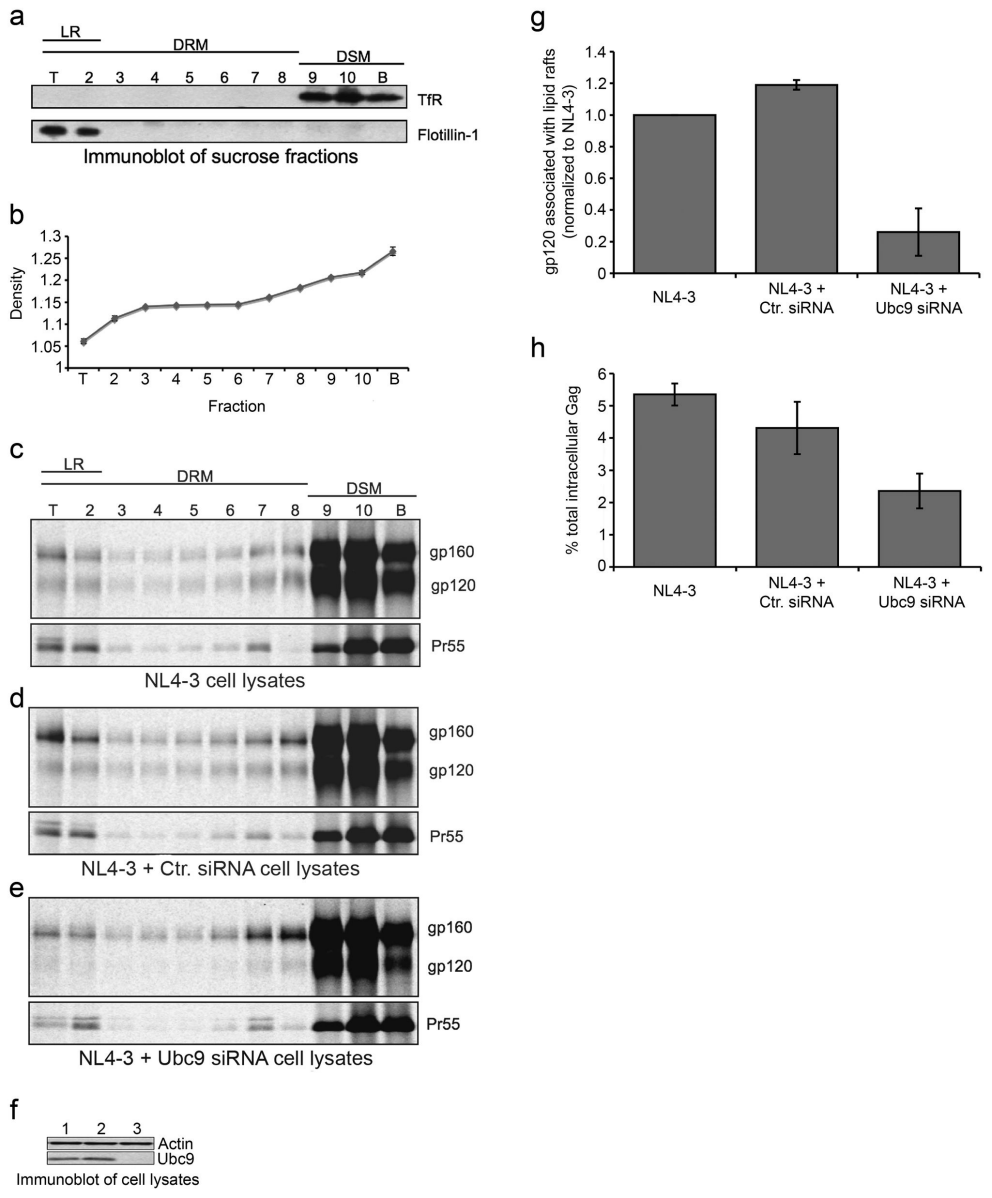
HIV-1 Gag and Env exhibit altered association with lipid rafts in Ubc9 knockdown cells. (a) Lipid raft isolation. Representative immunoblot of sucrose fractions for lipid raft markers Flotillin-1 and non-raft marker transferrin receptor 1 (TfR). Fractions containing lipid raft markers (LR), detergent soluble membranes (DSM; raft excluded), and detergent resistant membranes (DRM; non DSM). (b) Average density of all sucrose fractions prior to being adjusted to 1X lysis buffer. 293T cells were transfected with pNL4-3 alone (c), or in combination with either Ctr. siRNA (d) or Ubc9 siRNA (e). Cells were labeled with [^35^S] methionine/cysteine for 4 hours. Cells were placed on ice and transferred to a 4°C cold room where they were lysed with ice cold TNE buffer for 30 minutes on ice followed by homogenization with a 25G needle. Lysates were clarified in a cooled micro centrifuge and then adjusted to 60% sucrose and overlaid with a discontinuous sucrose gradient and centrifuged at 100,000 X g at 4C for at least 18 hrs. Gradients were fractioned into 11 equal samples using a Biocomp piston fractionator and adjusted to 1x lysis buffer. Viral proteins were immunoprecipitated with patient serum, separated by SDS PAGE, and visualized by phosphorimaging using The Discovery Series Quantity One software. Lipid raft floatation experiments were carried out in triplicate. Representative over-exposed gels are shown so that viral proteins associated with lipids rafts can be more easily visualized. (f) Immunoblot of transfected cell lysates; untransfected (lane 1), NL4-3 + Ctr. siRNA (lane 2), NL4-3 + Ubc9 siRNA (lane 3). (g) Ratio of gp120/Pr55 proteins associated with lipid rafts. (h) Percent of total cellular Gag associated with lipid rafts.

To more extensively examine the amount of Gag and Env associated with the PM during the assembly process we analyzed their association with lipid rafts (LR). Multiple lines of evidence have implicated that lipid rafts are important microdomains of the PM that facilitate virion assembly (reviewed in 95,96. To examine viral protein association to lipid rafts in Ubc9 knockdown cells, metabolic labeling combined with lipid raft floatation experiments were utilized. Transfected 293T cells were metabolically labeled, the cell lysates were fractioned, and analyzed for the presence or absence of viral and cellular proteins. Flotillin-1 is a protein marker of cellular LR and was only detected at the top of the gradient in fractions 1 and 2. Transferrin receptor (TfR), a protein excluded from detergent resistant membranes (DRM) and lipid rafts, was only detected in the bottom of the gradient in fractions 9, 10, and 11, indicating that there was a good separation between LR, DRM, and detergent soluble membranes (DSM) (Figure 5a). The densities of each of the fractions across all samples were assayed and showed that the gradients were consistent and reliably reproduced among samples (Figure 5b). DSM fractions from transfected cells were combined and immunoblotted for Ubc9 expression (Figure 5c). The majority of Gag and Env were detected in the DSM fractions, with very small amount found in the DRM and LR fractions regardless whether the cells were co-transfected with control siRNA, Ubc9 siRNA, or with NL4-3 alone (Figure 5d–f). In cells transfected with NL4-3, or in combination with control siRNA, gp160, gp120 and Gag were detected in the DSM and LR fractions. Viral proteins associated with DRM decreased as the sucrose density decreased towards the top of the gradient, then increased again when the fractions were enriched with LR. The ratios of Pr55 with gp160 and/or gp120 in LR fractions were similar between control cells. Interestingly, cells co-transfected with NL4-3 and Ubc9 siRNA showed altered levels of viral proteins associated with the DSM and LR factions. Ubc9 siRNA transfected cells showed about a 5-fold reduction in the amount of gp120 associated with LR fractions. However, similar LR Pr55/gp160 ratio was found when compared to control cells, suggesting that the amount of gp160 associated with the LR was not affected by Ubc9 expression levels even though there was a reduction in gp120 level (Figure 5g). The percentage of Gag associated with LR was also examined, as previous reports have suggested that Env trafficking to LR is dependent upon Gag co-trafficking to LR [97,98]. We found that in the absence of Ubc9 expression, Pr55 association with LR was decreased by almost 50% compared to the control cells (Figure 5h). We observed a good correlation between results obtained by three different technical approaches (i.e. membrane fractionation, floatation assay, and cell-surface envelope expression measurement by flow cytometry). In summary, our data suggests that Gag and Env trafficking and association with the plasma membrane is altered in the absence of Ubc9, and that gp120 stability is affected prior to the trafficking of gp120 to the PM and to the lipid raft microdomain in Ubc9 knockdown cells.

### Intracellular gp120 is degraded through the lysosomal pathway in the absence of Ubc9

To further understand how Env stability is affected in Ubc9 knockdown cells, we examined whether any of the predominant degradation pathways are involved. For these experiments we examined if proteasomal or lysosomal inhibitors will impede the degradation of mature Env in an effort to determine which degradative pathway was involved with Env degradation in Ubc9 knockdown cells.

**Figure 6:**
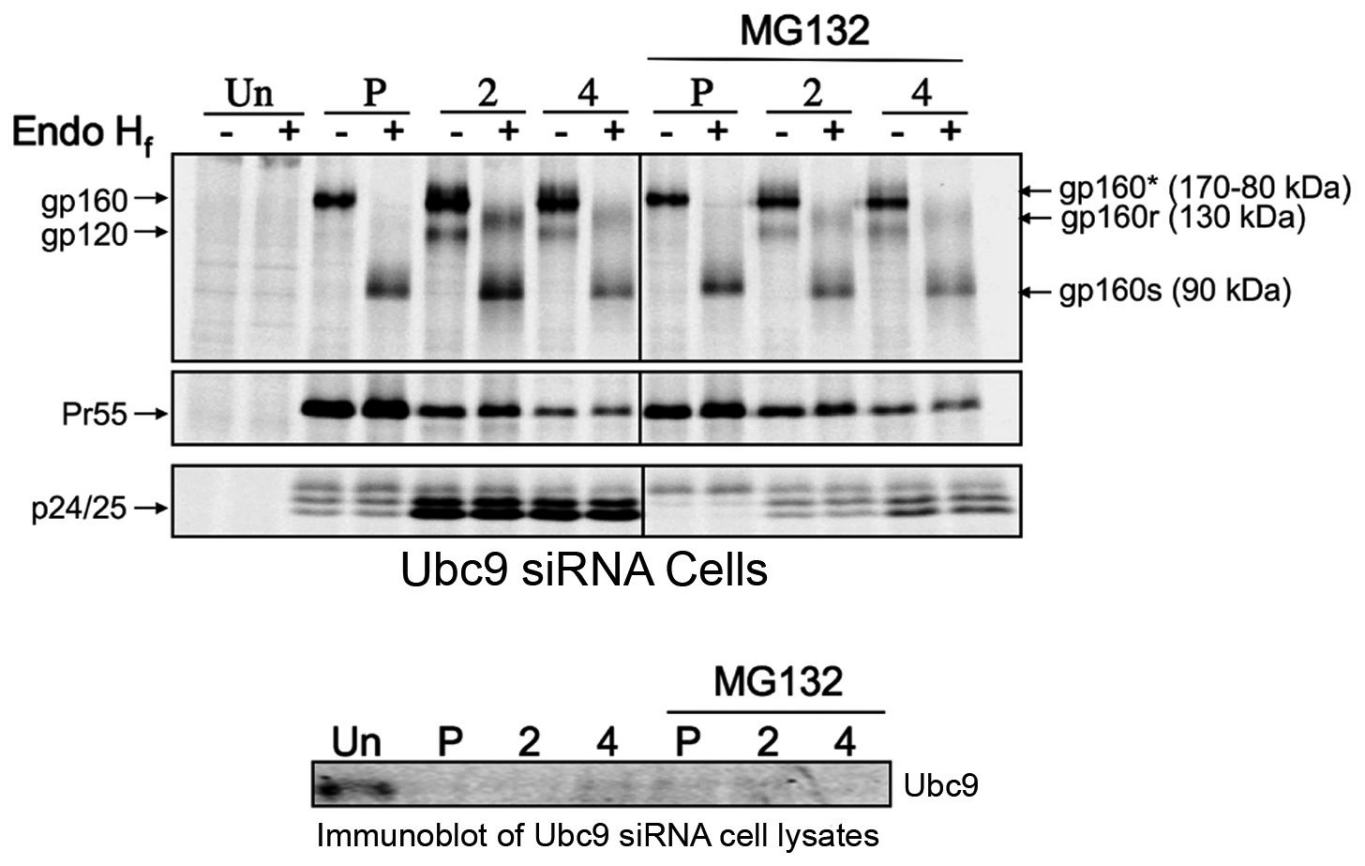
Env and Gag stability in Ubc9 knockdown cells in the presence of proteasome inhibitor MG132. 293T cells were transfected with Ubc9 siRNA and pNL4-3 as in previous experiments. MG132 (10µM) was added to the culture media for 1 hour prior to pulse chase experiments and was maintained throughout the experiment. Cells were pulse (P) labeled with [^35^S] methionine/cysteine for 1 hour and then chased for 2 and 4 hours. Cell and media associated viral proteins were solublized and immunoprecipitated with pooled AIDS patient sera, split equally, and incubated for 3.5 hours at 37°C in the presence, or absence of Endoglycosidase H _f_ (Endo H _f_). Samples were separated by SDS PAGE and visualized by phosphorimaging using The Discovery Series Quantity One software. A representative, over-exposed gel is shown so that partially Endo H_f_ resistant Env can be more easily visualized. Viral proteins and their positions in the gel are labeled on the left. The identity of Endo H_f_, untreated viral proteins and their positions in the gel are labeled on the right. Deglycosylated Endo H_f_ sensitive forms gp160 residing in the ER are labeled as gp160s. Partially deglycosylated, Endo H_f_ resistant forms of gp160 that have had their glycans modified in the TGN are labeled as gp160r. Forms of gp160 that have undergone glycan modification in the TGN but have not been cleaved are denoted as gp160* in Endo H_f_ untreated samples.

To examine the potential participation of the proteasomal degradation pathway MG132 (10µM) was used to inhibit the ubiquitin dependent 26S proteasome, and Env stability was assayed by pulse-chase experiments. We began by examining the proteasome as it has been previously demonstrated that the proteasomal pathway plays a crucial role during virion assembly and release [99]. Interestingly, no significant changes in Env maturation or stability was observed in the presence of the inhibitors, even though it has been reported by Bultmann et al. that a small fraction of Env is ubiquitinated and is degraded through the proteasome pathway [100]. In cells transfected with Ubc9 siRNA and NL4-3 in the presence of MG132, Gag displayed an assembly phenotype similar to what was described by Schubert et al. when cells were treated with proteasome inhibitors (Figure 6). Less Env and Gag proteins were expressed, Gag processing was slightly slower than in the untreated cells with less cell associated p24/25, and virion release was decreased by approximately 3.5-fold in the presence of MG132 (data not shown). Intracellular gp120 levels in Ubc9 siRNA cells did not appear to increase in the presence of MG132, suggesting that the proteasome is not the major degradation pathway involved in affecting the stability of intracellular gp120 in cells depleted of Ubc9.

**Figure 7:**
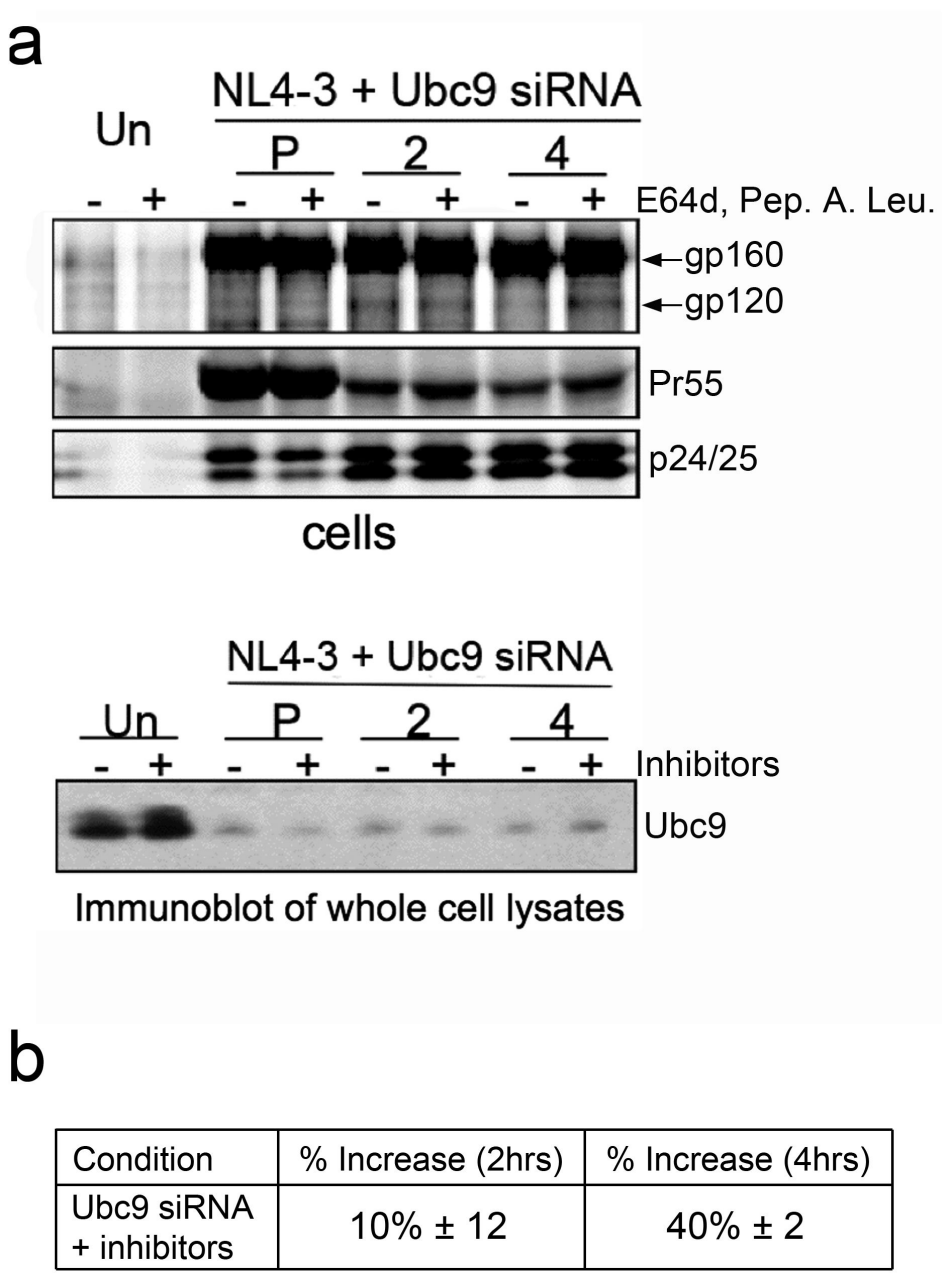
HIV-1 gp120 is degraded through the lysosomal pathway in Ubc9 knockdown cells. 293T cells were transfected with Ubc9 siRNA and NL4-3 as in previous experiments. (a) A combination of E64d (10µM), Pepstatin A (10µM), and Leupeptin (5µM), was added to the culture media 2 hours, or 45 minutes prior to pulse chase experiments. The amount of Pepstatin A was increased to 50 µM during the pulse chase experiment, while the amounts of E64d and Leupeptin were maintained at 10 µM and 5 µM respectively. Cells were pulse labeled with [^35^S] methionine/cysteine for 1 hour and then chased for 2 and 4 hours. Cell and media associated viral proteins were solublized and immunoprecipitated with pooled AIDS patient sera. Samples were separated by SDS PAGE and visualized by phosphorimaging using The Discovery Series Quantity One software. (b) Quantitation of increase in intracellular gp120 in Ubc9 siRNA knockdown cells compared to control cells.

The demonstration that the proteasome inhibitor MG132 was unable to restore intracellular Env levels, led us to further examine the potential role of the lysosomal degradation pathway. The lysosome pathway has been previously implicated as having a role in HIV-1 Env degradation and virion assembly. A combination of specific lysosomal protease inhibitors, E64d, Pepstatin A, and Leupeptin [101–103] were used to determine if lysosomal degradation was involved in Env degradation in Ubc9-depleted cells (Figure 7). In the presence of these lysosome inhibitors, there were 40% more intracellular gp120 at the 4-hour chase time as compared to untreated Ubc9 knocked down cells (Figure 7). Immunoblots of intracellular Env following longer inhibitor treatments were also in agreement with pulse chase data using lysosomal inhibitors (data not shown), suggesting that the lysosome degradation is likely to be involved in the depletion of intracellular gp120 in Ubc9 knockdown cells.

## Discussion

In this study we have continued to decipher the role of Ubc9 on HIV-1 assembly, and to further understand how Ubc9 and Gag contribute to intracellular Env stability and incorporation into assembling virions. We found that in Ubc9 knockdown cells the decrease in intracellular gp120 levels is due to increased degradation of mature Env in the lysosome at a post-TGN trafficking step. Analysis of Env trafficking to the PM by various assays suggests that degradation of mature Env likely occurs before insertion into the PM. Lysosome inhibitors partially restored mature intracellular Env levels.

**Figure 8:**
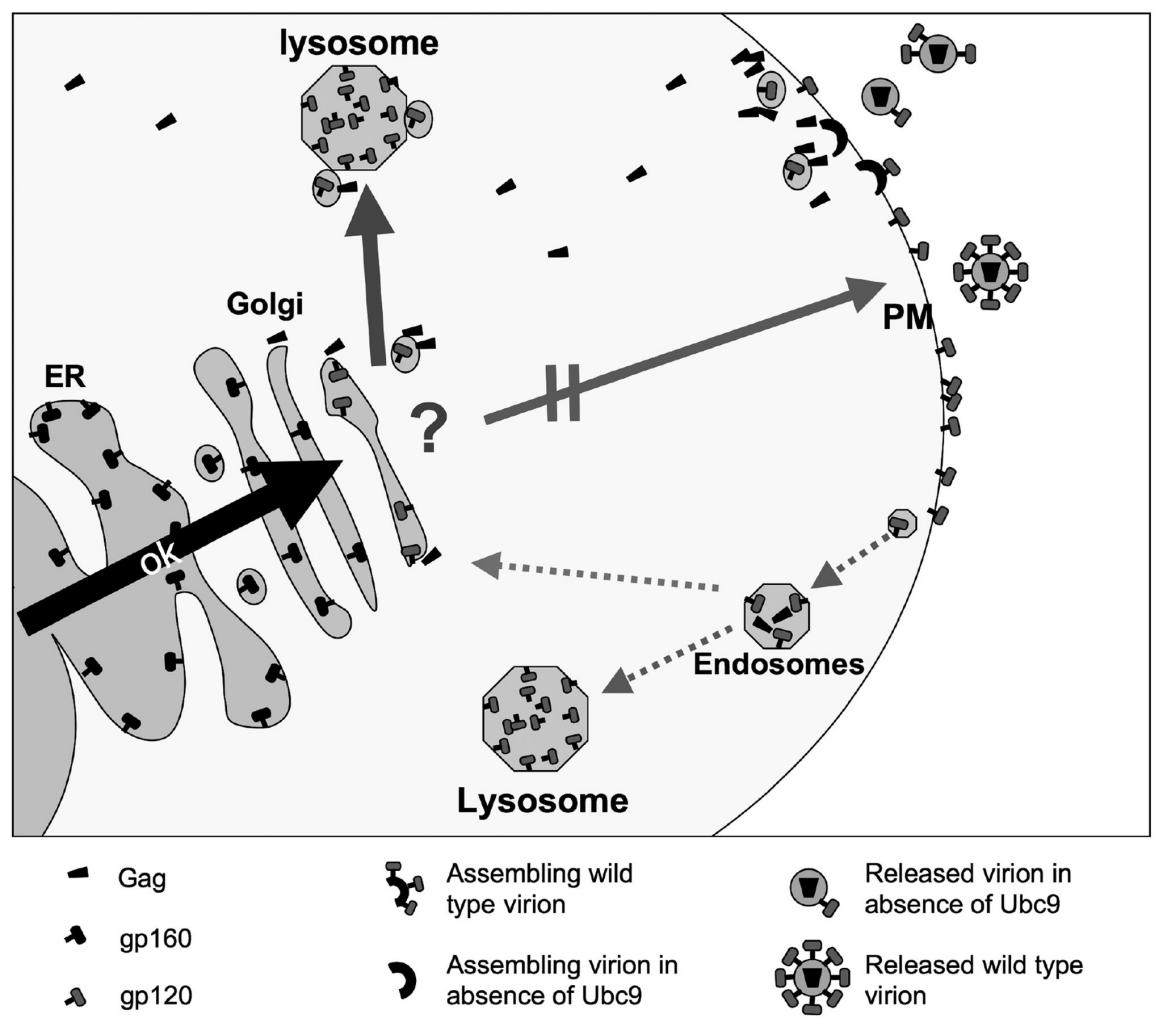
Working model of Env trafficking through the cell in Ubc9 knockdown cells. HIV-1 Env trafficking from the ER to the TGN occurs normally. After cleavage into gp120/gp41 and transport out of the TGN, Env is targeted for degradation in the lysosome instead of the assembly site of the plasma membrane. This mistrafficking of Env for degradation could be due to altered interactions with Gag, as Gag itself is mistrafficked to the assembly site in Ubc9 knockdown cells. Another potential mechanism is that as Gag is mistrafficked to the assembly site in Ubc9 knockdown cells, it may not stabilize Env at the plasma membrane causing Env to be quickly endocytosed and degraded. In Ubc9 knockdown cells, a portion of endocytosed Env may then be targeted for degradation in the lysosome instead of recycling back to the TGN. Alternatively, the trafficking or function of yet unknown cellular factor(s) that are involved in Env stability and packaging into assembling virions may have been disrupted possibly altering Gag/Ubc9 interactions (question mark).

Based on our results, we hypothesize two potential mechanisms that may explain how interrupting the interaction between Gag and the cellular host factor Ubc9 may lead to gp120/gp41 degradation and decreased incorporation into the assembling virions (Figure 8). In the first model, Gag intracellular trafficking is affected due to the Ubc9-depletion. The change in Gag trafficking alters the Env-Gag interaction to cause mature Env to be mistargeted for degradation before its transport to the PM. The second model proposes that depletion of Ubc9 diminishes the ability of Gag to stabilize Env on the PM, leading Env to be quickly endocytosed and targeted for degradation in the lysosome instead of recycling in the TGN [4].

Our results favor the first model. The altered Gag-Env interaction in Ubc9 knockdown cells may directly or indirectly led to the mistargeting of the Env containing secretory vesicles for degradation before trafficking to the PM. In Ubc9-depleted cells, Gag displayed altered trafficking and association with lipid rafts, a microdomain important during the very late stages of virion assembly (reviewed in 95,96. We have previously observed that Gag co-localized with Ubc9 in close proximity to the PM, and suggests that this interaction may be important during the later stages of virion assembly [49]. However, the earlier stages of Gag trafficking may have also been disrupted since Ubc9 was identified as a host factor localized to the Gag perinuclear clusters (GPC), which were hypothesized to be the early intermediate Gag trafficking sites [18]. When Ubc9 is not present at the GPC, Gag may not be targeted correctly through the cytoplasm, and affects its interaction with other viral and cellular factors during virion assembly.

Env and Gag have been hypothesized to first interact and influence each other at an intracellular site, at or near the TGN or recycling endosomes [19,26,27,43]. It is possible that the interruption of normal Gag-Ubc9 interactions in the Ubc9–depleted cells may also affect Gag-Env interactions to lead to a disruption of their co-trafficking to the site of assembly. This disruption could lead to Env being mistrafficked/sorted and targeted for degradation instead of trafficking to the normal virion assembly site. This is supported by reports implicating Ubc9 involvement in TGN protein sorting, leading to aberrant trafficking within the cell. Studies with GLUT4, an insulin-regulated glucose transporter have shown that Ubc9 plays a role in regulating GLUT4 stability and trafficking [57]. GLUT4 expression was decreased by approximately 50% in Ubc9-depleted cells due to an increase in its degradation. It was hypothesized by Liu et al. that Ubc9 may interact with the Arf-binding proteins (GGA), whose function is to sort proteins moving through the secretory pathway and are involved with the formation of GLUT4 storage vesicles. Their results suggest that Ubc9 may function to negatively regulate the trafficking of secretory vesicles from the TGN to sites of degradation [57]. Interestingly, GGA and Arf proteins were shown by Joshi et al. to be important host factors that regulate HIV-1 assembly and release, and GGA2 appeared to play a role in Env maturation [29]. However, our data indicate that the Ubc9 knockdown mediated decrease of intracellular levels of gp120 does not appear to be via the same mechanism, as we have not observed changes in gp160 processing as was reported with GGA2 over-expression.

It is also possible that Gag and Ubc9 are part of a larger complex that is not yet characterized, and disruption of the normal Ubc9-Gag interaction interferes with the normal function or composition of this complex. Disruption of this complex may result in the mistrafficking/sorting of Env to increase the degradation of Env in the lysosome. In this complex, Gag may be “tethering” and retaining Ubc9 in close proximity to other proteins within the complex to increase their interaction with Ubc9. For example, it has been previously reported that interactions between cellular factor p14^ARF^ and Ubc9 enhanced the SUMOylation of p14^ARF^ binding partners. This demonstrated that Ubc9 binding to cellular factors may act as a “tether” to retain the SUMO-Ubc9 complex in close proximity to unmodified target proteins in order to increase the efficiency by which they are SUMOylated [104]. A similar model has been proposed for dynamin and SUMOylation mediated endocytosis [105].

Although we had previously reported that over-expression of the catalytically inactive trans-dominant negative Ubc9 mutant (C93A) did not lead to a decrease in infectivity of the HIV-1 virion produced, a total block in SUMOylation was unlikely [49], and it is possible that only a very low level of SUMOylation is needed during the assembly process. Interestingly, Zamborlini et al. demonstrated that HIV-1 Gag-Pol cleavage intermediates are SUMOylated with SUMO-2 in a cell line that over-expressed SUMO-2. However, mutations of the SUMO acceptor sites within integrase did not affect the enzymatic activity of integrase or virion assembly. This suggests that if SUMOylation of Gag-Pol plays a role during assembly it may be at an early assembly event and must be transient since SUMO or SUMOylated forms of Gag, or integrase have not been detected under normal conditions [106–108].

Our data cannot completely rule out the alternative model involving endocytosis since the SUMOylation pathway has been previously reported to be involved in the endocytosis of cellular proteins [105,109]. We observed changes in Gag trafficking to lipid rafts in Ubc9 knockdown cells, which may affect Env stability on the PM due to changes in Gag/Env interactions and Env recruitment to lipid rafts. Our attempts to block endocytosis using the commonly used endocytosis inhibitor Chlorpromazine was unsuccessful because we found that the treatment also blocked Env transport to the TGN from the ER (data not shown). However, we do not believe that altered endocytosis of Env, directly or indirectly, is the primary mechanism leading to increased intracellular Env degradation in the lysosome. It has been shown that point mutations in either MA or the CT of Env, that disrupt Env incorporation by altering their direct or indirect interactions at the site of assembly, did not decrease the intracellular levels of Env as was observed in our Ubc9 knockdown cells [97,110–113]. Furthermore, knockdown of Tip47, a host protein reported to be involved in Env incorporation and retrograde transport to the TGN from the PM, did not result in decreased intracellular Env levels [4,26,27]. In addition we did not observe changes in intracellular levels of host cell surface proteins E-cadherin (Figure 3) and TfR (data not shown), both of which also undergo AP-2/clathrin dependent endocytosis [114–116]. Our data thus suggests that endocytosis dependent degradation in Ubc9 knockdown cells is not upregulated, and is in agreement with what others have reported with Ubc9 knockdown [109].

Interestingly, the matrix mutant (HXB2WANEO) has been described to affect mature intracellular levels of Env in a very similar manner to what we have described here [48]. This similarity suggests that a disruption in MA and Env CT interactions in Ubc9-depleted cells could lead to enhanced Env degradation. However, other mutations within MA or Env CT that have also been described to block Env incorporation did not seem to affect the intracellular Env levels, or Env trafficking to the PM [110–113]. Since the Env of these mutants presumably undergo increased endocytosis from the PM due to the interruption of MA/CT. These studies suggest that the decreased levels of intracellular gp120 levels in Ubc9-depleted cells we observed is not due to endocytosis, and is likely due to a mechanism that is distinct from the other MA/CT mutants that disrupt Gag/Env interactions. Furthermore, in the absence of Gag expression, Env does not exhibit decreased intracellular levels. This would be observed in Ubc9-depleted cells if degradation following endocytosis was responsible for reduced levels of intracellular mature Env, or if Ubc9 acts directly with Env to regulate its trafficking and stability. Taken together, increased endocytosis due to the inability of Gag to stabilize Env at the PM, or non-specific endocytosis is not likely to be the primary mechanism for decreased Env stability in Ubc9 depleted cells.

Extensive studies have been carried out to characterize the functions of Env CT using point mutations and deletions, however the precise molecular mechanisms of how Gag and Env coordinate the assembly of infectious virions on the plasma membrane remains elusive (Reviewed in 7–10. Interestingly, careful characterization of a panel of CT deleted HIV-1 Env mutants displayed defects in almost every functional assay with the exception that mutant gp41 was found to be incorporated at a higher level with extracellular virions than with WT. However, less gp120 was co-incorporated with the mutant gp41 [117]. Based on our data, one might hypothesize that this mutant my be resistant to Ubc9 knockdown based on our data and hypothesis, however this mutant displays multiple defects in processing, stability, and trafficking making it difficult to analyze the domains involved upon depletion of Ubc9 in HIV-1 producer cells. Future studies will be needed to more closely examine and identify which regions and amino acids that are required for the Env degradation phenotype in Ubc9 depleted cells.

In conclusion, our study further support that Ubc9, through interactions with Gag, influence the stability and trafficking of mature HIV-1 Env to the site of assembly and ultimately affects the amount of mature Env incorporated into released virions. This data provides further insight as to how Ubc9 is involved in HIV-1 assembly and could provide new strategies for antiviral drug development.
